# The Acceptability of a Rolling Compassion Focused Therapy Group Embedded Within an Eating Disorder Day Hospital Programme: A Brief Report

**DOI:** 10.1002/erv.70053

**Published:** 2025-11-06

**Authors:** Conal Twomey, Olivia Cagney, Fionnuala McEnerny, Clodagh Dowling

**Affiliations:** ^1^ Psychology Department St Patrick's Mental Health Services Dublin Ireland; ^2^ School of Psychology University College Dublin Dublin Ireland

**Keywords:** acceptability, compassion focused therapy, day hospital, eating disorders

## Abstract

**Purpose:**

Interventions embedded within eating disorder day hospital programmes are often provided on a rolling basis with flexible entry and exit points. This clinical audit examined the acceptability of one such intervention: a rolling *Introduction to Compassion Focused Therapy for Eating Disorders* (CFT‐E) group.

**Methods:**

Acceptability was assessed across 22 sessions, over 5 months. Using anonymous feedback forms, participants rated each session using Likert scales and open‐ended responses.

**Results:**

Across 112 total attendances, session ratings indicated good acceptability, particularly for improving motivation, knowledge of compassion‐focused principles, and personal and supportive group connections (all mean scores > 4.0). The trajectory of the mean ratings across each session also pointed to consistent and enduring acceptability. CFT‐E theory (*n* = 62) was the most frequently reported helpful category, followed by group process factors (*n* = 47), and improved self‐awareness (*n* = 9). Openness from the group, particularly regarding discussing eating disorders, was referred to in a positive light across various analyses. Some engagement issues (*n* = 12) were also highlighted.

**Conclusions:**

The demonstrated acceptability of this rolling *Introduction to CFT‐E* group supports its continued provision and justifies further evaluation of similar introductory, compassion‐focused formats in eating‐disorder services. Engagement issues offer room for improvement.

## Highlights

- This encompasses the first investigation of the acceptability of a *rolling Introduction to CFT‐E* group embedded within an eating disorder day‐hospital programme.
- Consistently high acceptability ratings and positive qualitative feedback indicate that the group was well received across weekly sessions over a 5‐month period.
- The demonstrated acceptability may encourage the further investigation of similar rolling CFT‐E groups, with clinical services secure in the knowledge that acceptability is unlikely to be a major cause for concern.

## 1 Introduction

The association of shame with eating disorder (ED) behaviours has been consistently demonstrated (Nechita et al. 2021). In compassion focused therapy for EDs (CFT‐E), it has been hypothesised that ED behaviours are maladaptive attempts to regulate shame and related self‐criticism through emotion distancing, distraction, and/or pride functions (Goss and Allan 2009). With the option to incorporate behavioural interventions for EDs, CFT‐E targets shame and self‐criticism through the cultivation of self‐compassion, within a multi‐faceted group intervention primarily consisting of psychoeducation informed by evolutionary theory, formulation, self‐soothing and imagery techniques, compassionate motivation enhancement, and social connection activities (Goss and Kelly 2022).

Reviews have yielded support for CFT‐E's effectiveness for increases in compassion‐related outcomes and reductions in shame and eating pathology (Basran et al. 2022; Millard et al. 2023). In addition, a recent randomized controlled trial in an intensive treatment setting reported comparable outcomes for CFT‐E and cognitive behavioural therapy, with CFT‐E showing superior maintenance of gains at 1‐year follow‐up among participants with trauma histories (Vrabel et al. 2024). The flexibility of CFT‐E is also noteworthy: documented interventions range from 4 to 20 sessions; the approach can function as either a standalone or adjunctive intervention; feasibility studies support both online and in‐person delivery; and it has been applied to both transdiagnostic and diagnosis‐specific populations (Basran et al. 2022; Goss and Kelly 2022; Marques et al. 2024). Further flexibility is evident in the development of rolling CFT groups across other clinical contexts, including acute inpatient care and work with individuals experiencing personality‐related difficulties (Heriot‐Maitland et al. 2014; Lucre 2022).

Building on this emerging empirical support and established delivery format flexibility, a rolling *Introduction to CFT‐E* group intervention was implemented within the ED day hospital programme at an independent, not‐for‐profit mental health service in Ireland. The finding of an association of reduced shame early in treatment with better ED outcomes (Kelly et al. 2014) was another motivating factor in the provision of the group within this setting.

Service users—with varying ED diagnoses—typically attend the ED day hospital programme for 8 weeks, yet they enter and exit treatment at different time points. Therefore, embedded group interventions are delivered on a rolling basis to accommodate service users at different stages of their recovery journey. The requirement for a rolling group had two main implications for planning *Introduction to CFT‐E*: (1) each session needed to be accessible to both beginners and more experienced participants; and (2) although guided by an introductory‐level CFT‐E theoretical framework, topics of group discussions were—to some degree—adapted based on the perceived readiness of specific group cohorts. Of note, the rolling group can be considered an adjunctive version of CFT‐E—previous adjunctive versions have added elements of CFT theory to comprehensive cognitive behavioural therapy (CBT) programmes, while *Introduction to CFT‐E* adds elements of CFT to the ED day hospital programme which itself addresses various areas such as self‐esteem, goal‐setting, body image, meal‐planning, weighing, and CBT skills.

Within the above context, the main clinical aims of the *Introduction to CFT‐E* group are to familiarize participants with key compassion‐focused principles relevant to eating disorders, particularly those targeting shame and self‐criticism. The group also seeks to increase participants' motivation to engage in reflective psychological work, introduce compassion‐based skills to support emotional awareness and regulation, enhance comfort in discussing emotional and eating‐related experiences, and foster the development of personal and supportive group connections.

In practice, the group serves multiple functions within the wider treatment pathway. It is not necessarily a precursor to a full CFT‐E programme but provides participants with an experience of compassion‐focused principles that can inform ongoing psychological work and future therapeutic engagement. For some, it serves as a brief, stand‐alone intervention during their day hospital participation; for others, it contributes to onward care planning, including potential referral to CFT‐E and other outpatient group programmes such as transdiagnostic CFT, Group Radical Openness, Dialectical Behaviour Therapy, and Schema Therapy. It also offers clinicians insight into participants' readiness for further group work and the suitability of compassion‐focused approaches for individual needs.

Given the necessity to provide the *Introduction to CFT‐E* group on a rolling basis, there was uncertainty around how well the group intervention would be received by service users. A clinical audit was therefore undertaken to assess the group's acceptability in relation to its aims. After each session of the *Introduction to CFT‐E* group over a five‐month period in 2022, anonymised self‐report data were collected using brief questionnaires assessing motivation, knowledge of compassion‐focused principles, group connections, comfort with open discussion, and perceived skills development. We also aimed to generate findings relevant to future evaluations.

## 2 Methods

### 2.1 Participants and Procedure

Participants were service users—diagnosed with anorexia nervosa, bulimia nervosa, binge eating disorder, and other specified eating disorders—attending the ED day hospital programme at SPMHS. Data were collected through brief anonymous questionnaires administered at the end of each session. Data were collected across 22 weekly sessions—the initial aim was for 26 weeks (6 months) but staffing and issues curtailed the time period somewhat.

### 2.2 Intervention

Facilitated by two clinical psychologists and an assistant psychologist, the rolling *Introduction to CFT‐E* group is 2.5 h in duration, including a 30‐min midway break. Facilitators are required to complete certified CFT‐E training and receive monthly supervision from a senior clinician with extensive experience in CFT‐E delivery. The group begins with a soothing and/or imagery practice, with such practices based on established CFT exercises (Gilbert 2010a, 2010b). In early iterations of the programme, a minority of participants reported finding the soothing and imagery exercises initially uncomfortable, often due to self‐criticism or difficulties with emotional regulation. Based on this clinical observation, to promote safety and engagement, brief ‘ice‐breakers’ and other warm‐up techniques are sometimes used at the start of *Introduction to CFT‐E* sessions. Examples include discussing the origins of participants' names, a light‐hearted ping‐pong activity emphasizing cooperation and playfulness, and reflections on the perceived qualities of compassion (prior to psychoeducation around them).

Following these initial practices, psychoeducation is provided around a CFT‐E concept, such as evolutionally‐grounded emotion regulation systems, the ‘tricky brain’, or the qualities of compassion (Gilbert 2014). Afterwards, participants are invited to reflect on the psychoeducation through group discussions, worksheet exercises, videos, and whiteboard work. Participants then apply the chosen concept to their personal experiences—both in relation to their ED behaviours and more generally. A common theme of discussion encompasses ‘fears, blocks, and resistances’ to compassion (Gilbert et al. 2011), which often can be linked to ED‐related cognitions and behaviours. Reflections on motivation, therapeutic progress, and recovery‐related goals also tend to arise frequently.

Of note, the *Introduction to CFT‐E* group follows a recurring suite of core topics delivered on a rolling basis rather than as a fixed course. Core sessions include the ‘tricky brain’ and unhelpful loops, evolutionarily grounded emotion regulation systems (the ‘three circles’), compassionate imagery, the definition and qualities of compassion, fears, blocks, and resistances to compassion, and the functional analysis of the inner critic (Gilbert 2010a, 2010b, 2014). The three‐circle model may be covered as a single session or explored in greater depth across separate sessions with greater emphasis given to the threat, drive, and soothing systems. Facilitators choose and adapt topics based on participants' previous attendance, group readiness, and clinical presentation, ensuring exposure to key CFT‐E principles irrespective of entry point.

### 2.3 Measures

All scales/questions are listed in the Results. The anonymised acceptability questionnaire had five‐point scale items asking participants how they would rate the just‐completed session in five areas. Qualitatively, participants were also asked two open‐ended questions in relation to the helpfulness of the session, and other feedback.

### 2.4 Data Analysis

Acceptability ratings for each session and across all sessions were analysed using descriptive statistics. For the qualitative feedback, open‐ended responses were analysed using content analysis which presented categorised textual data in the form of frequencies (Krippendorff 2019). Here, the second author constructed categories based on an initial review of the data and these categories were reviewed by the first author, and collaboratively edited. Next, the second author coded the data into the categories, before review by the first author. Qualitative feedback responses were analysed descriptively at the response level (rather than by individual participant) to capture the range and frequency of themes mentioned across sessions. This approach was chosen because the group operated in a rolling format, meaning participants could attend varying numbers and combinations of sessions and provide feedback after each one. Analysing data at the response level therefore allowed each session's unique content and participant experience to be represented within the overall analysis.

## 3 Results

### 3.1 Group Attendance

The number of participants attending at least one session of *Introduction to CFT‐E* was 35, with a mean of 3.2 sessions attended (SD = 2.32; range = 1–12). Eleven participants (31%) attended only one session, yet their data were included in analyses in line with the rolling nature of the group. Across the 22 sessions, the total number of attendances (including repeat participation) was 112. The mean group size per session was 5.2 (SD = 1.60; range = 3–9). As per general clinical audit practice, demographic data is not reported beyond the broad diagnostic information already stated in the Methods.

### 3.2 Acceptability of *Introduction to CFT‐E*: Group Mean Rating Scores Across All 22 Sessions

The mean session ratings (out of 5) across all 22 sessions were as follows:

1. Increasing your motivation to do the next session of the group: *M* = 4.2, SD = 0.98.
2. Increasing your knowledge of compassion‐focused therapy: *M* = 4.1, SD = 0.93.
3. Developing personal and supportive connections with other group members: *M* = 4.0, SD = 1.05.
4. Increasing comfort with talking openly about eating disorders: *M* = 3.9, SD = 1.16.
5. Learning skills relevant to your ED recovery: *M* = 3.8, SD = 1.03.

### 3.3 Acceptability of *Introduction to CFT‐E*: Trajectory of Group Mean Rating Scores Across All 22 Sessions

**FIGURE 1:**
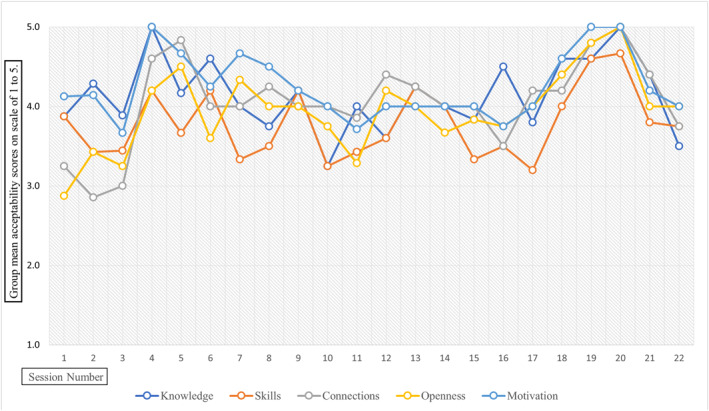
Group mean acceptability scores across 22 sessions of *Introduction to CFT‐E.* Each line represents the group's mean rating per session (out of 5) for one of the following feedback domains: knowledge, skills, connections, openness, and motivation.

Figure 1 displays the trajectory of group mean ratings (out of 5) for each of the five feedback domains—knowledge, skills, connections, openness, and motivation—across the 22 sessions. The lowest group mean score was in Session 2 for the ‘connections’ scale (2.9). The only other time the score dropped below 3.0 was for the ‘talking openly’ scale in Session 1 (2.9). The maximum score of 5.0 was indicated on seven occasions: in Session 4 for the ‘knowledge’ and ‘motivation’ scales; in Session 19 for the ‘motivation’ scale; and in Session 20 for the ‘knowledge’, ‘connections’, ‘talking openly’, and ‘motivations’ scales.

### 3.4 Acceptability of *Introduction to CFT‐E*: Qualitative Feedback

Frequencies (*n*) reported below refer to the number of responses coded under each theme, rather than the number of unique participants. In response to ‘What was most helpful from today's session and why?’, the category of CFT‐E theory was most frequently reported as most helpful (*n* = 62). Its sub‐categories were ‘three systems’ theory (*n* = 14), the definition/qualities of compassion (*n* = 9), visual imagery (*n* = 9), the ‘tricky brain’ (*n* = 9), breathing exercises (*n* = 7), the ‘self‐critic’ (*n* = 6), CFT‐E‐relevant case studies (*n* = 5), and using the senses *(n* = 3). The next most frequent category was group process (*n* = 47). Its sub‐categories were open group discussions (*n* = 28), small groups (*n* = 9), games (*n* = 4), feeling safe (*n* = 4), and being given time to speak (*n* = 2). A self‐awareness category was also highlighted as helpful, with less frequency (*n* = 9).

In response to ‘Do you have any other feedback (e.g., what was helpful or unhelpful, or how to improve the session)?’, the most frequent category was positive feedback (*n* = 16). Its sub‐categories were overall helpfulness (*n* = 5), openness from the group (*n* = 5), visual and written materials (*n* = 3), and space to reflect (*n* = 3). The next most frequent category was engagement issues (*n* = 12). Its sub‐categories were desire for more group interaction (*n* = 7), and attentional barriers (*n* = 5). The remaining categories were miscellaneous negative feedback (*n* = 6) and suggested improvements to the programme (*n* = 4). Further information regarding the above analyses, including illustrative quotes/examples, is available in Table S1.

## 4 Discussion

### 4.1 Summary of Main Results

Based on a total attendance of 112 across 22 sessions, session ratings illustrated good acceptability of the rolling *Introduction to CFT‐E* group, particularly relating to motivation, knowledge of CFT, and personal and supportive connections, with slightly less—but still good—acceptability in relation to comfort with talking openly about EDs, and learning relevant skills. The trajectory of group mean ratings across the 22 sessions indicated consistent acceptability over time. Across all 110 mean scale ratings calculated for the five acceptability domains, scores fell below 3.0 (out of 5) only twice (1.8%). Overall, this pattern suggests that participants reported a stable and generally positive experience of the *Introduction to CFT‐E* group throughout the audit period. This effect is best appreciated visually in Figure 1, which illustrates the stability and consistency of the group's mean acceptability ratings across sessions.

Qualitative feedback revealed that CFT‐E theory (particularly regarding the ‘three systems' of emotional regulation) was the most frequently reported helpful category across each session—this corresponds with the high mean session rating for knowledge of CFT (4.0). Group process factors (particularly regarding open group discussions) were also frequently reported as most helpful, corresponding with the mean session rating of 3.9 for talking openly about EDs. Furthermore, openness from the group also emerged as a sub‐category under the ‘other feedback’ question.

Self‐awareness increases were occasionally reported as helpful. This corresponds with positive feedback relating to ‘space to reflect’, under the ‘other feedback’ question. Another category to emerge under the ‘other feedback’ question was engagement issues, with a desire for more group interaction and attentional barriers as sub‐categories.

### 4.2 Comparison With Other Findings

Although not directly comparable, the findings supporting the acceptability of the *Introduction to CFT‐E* group are in line with the emerging evidence‐base for CFT‐E (Basran et al. 2022; Millard et al. 2023; Vrabel et al. 2024). The findings also support the flexibility in CFT‐E's delivery, here demonstrated within a lower intensity format that has yielded support elsewhere (Basran et al. 2022; Goss and Kelly 2022; Marques et al. 2024) and also in a rolling format previously shown to be acceptable for CFT programmes in non‐ED populations (Heriot‐Maitland et al. 2014; Lucre 2022). The consistently positive trajectory of acceptability across sessions, despite the diversity of CFT concepts covered, further highlights the model's adaptability to varied clinical contexts.

### 4.3 Implications for Clinical Practice and Future Research

The demonstrated acceptability of the *Introduction to CFT‐E* group supports its continued provision and its more stringent future evaluation. The findings are suggestive of various clinical outcomes that are worthy of investigation, including self‐compassion, motivation to change, de‐shaming and open dialogue around ED‐related experiences, and increased self‐awareness. The de‐shaming outcome is particularly worthy of investigation given the previously indicated link between reduced shame early in treatment with better ED outcomes (Kelly et al. 2014). The acceptability of CFT‐E *theory* may encourage the delivery of similar rolling groups in ED day hospital settings.

The highlighted engagement issues could form the basis of a follow‐up investigation and/or evaluation of ways to address them. Desire for more group interaction could be addressed though more open discussion around the impacts of limited interaction, and scaffolding techniques such as writing thoughts down before sharing in the wider group, small groups exercises, and space for the group to converse in the temporary absence of facilitators. Attentional issues could be addressed by reviewing factors such as the (afternoon) time of the group, its duration, and the types of activities involved. Previously identified barriers to engagement with ED interventions (Ali et al. 2017) such as shame/stigma, and reduced motivation are also relevant.

Regarding other future research avenues, service evaluations could usefully examine participants' post‐programme care pathways to explore whether engagement in the *Introduction to CFT‐E* group influences subsequent participation in CFT‐E or other therapeutic interventions. Such work would help clarify the longer‐term role of introductory compassion‐focused groups within eating‐disorder treatment programmes. While this clinical audit focused on service‐user feedback, future evaluations could also incorporate clinician reflections on the process of delivering a rolling CFT‐E group. Such perspectives would help to contextualise acceptability findings, inform facilitator training and supervision needs, and contribute to the refinement of implementation strategies in intensive treatment settings.

### 4.4 Strengths and Limits

Owing to the rarity of rolling CFT‐E programmes, the findings are arguably novel and informative. The study was bolstered by the collection of routine clinical data over a 5‐month period. Mixed methodology allowed the findings to be explored from different angles. Regarding limits, the absence of demographic data restricts generalisability. Rating scales were brief and unstandardised. The feedback questionnaire was short, but this promoted engagement and minimised disruption to the group.

## 5 Conclusion

Consistent acceptability ratings and positive qualitative feedback suggest that an introductory, lower‐intensity compassion‐focused format can be meaningfully integrated into day hospital programmes. These findings extend the growing evidence base for CFT‐E by highlighting its flexibility of delivery—here demonstrated through a rolling, adjunctive format that introduces core compassion‐focused principles to support engagement, emotional awareness, and recovery‐oriented reflection within day hospital care. The demonstrated acceptability may encourage the further investigation of similar rolling CFT‐E groups, with clinical services secure in the knowledge that acceptability is unlikely to be a major cause for concern. Future research could usefully examine the clinical impact and longer‐term outcomes of such introductory compassion‐focused interventions within diverse treatment settings.

## Ethics Statement

The study was approved by SPMHS as a clinical audit.

## Conflicts of Interest

The authors declare no conflicts of interest.

## Supporting information


**Table S1:** Findings from quantitative content analysis of open‐ended responses to two questions.

## Data Availability

Research data are not shared for reasons of confidentiality.

## References

[erv70053-bib-0001] Ali, K. , L. Farrer , D. B. Fassnacht , A. Gulliver , S. Bauer , and K. M. Griffiths . 2017. “Perceived Barriers and Facilitators Towards Help‐Seeking for Eating Disorders: A Systematic Review.” International Journal of Eating Disorders 50, no. 1: 9–21. 10.1002/eat.22598. 27526643

[erv70053-bib-0002] Basran, J. , J. Raven , and P. Plowright . 2022. “Overview of Outcome Research on Compassion Focused Therapy: A Scoping Review.” In Compassion Focused Therapy: Clinical Practice and Applications, edited by P. Gilbert and G. Simos , 1 ed., 600–615. Routledge. 10.4324/9781003035879.

[erv70053-bib-0003] Gilbert, P. 2010a. Compassion Focused Therapy: Distinctive Features. 1St Ed. Routledge. 10.4324/9780203851197.

[erv70053-bib-0004] Gilbert, P. 2010b. Training Our Minds In, With and for Compassion. an Introduction to Concepts and Compassion‐Focused Exercises. Compassionate Mind Foundation.

[erv70053-bib-0005] Gilbert, P. 2014. “The Origins and Nature of Compassion Focused Therapy.” British Journal of Clinical Psychology 53, no. 1: 6–41. 10.1111/bjc.12043. 24588760

[erv70053-bib-0006] Gilbert, P. , K. McEwan , M. Matos , and A. Rivis . 2011. “Fears of Compassion: Development of Three Self‐Report Measures.” Psychology and Psychotherapy: Theory, Research and Practice 84, no. 3: 239–255. 10.1348/147608310x526511. 22903867

[erv70053-bib-0007] Goss, K. , and S. Allan . 2009. “Shame, Pride and Eating Disorders.” Clinical Psychology & Psychotherapy 16, no. 4: 303–316. 10.1002/cpp.627. 19639646

[erv70053-bib-0008] Goss, K. , and A. Kelly . 2022. “The Roles of Shame, Self‐Criticism, and Compassion Focused Therapy in Eating Disorders and Disordered Eating.” In Compassion Focused Therapy: Clinical Practice and Applications, edited by P. Gilbert and G. Simos , 1 ed., 519–533. Routledge. 10.4324/9781003035879.

[erv70053-bib-0009] Heriot‐Maitland, C. , J. B. Vidal , S. Ball , and C. Irons . 2014. “A Compassionate‐Focused Therapy Group Approach for Acute Inpatients: Feasibility, Initial Pilot Outcome Data, and Recommendations.” British Journal of Clinical Psychology 53, no. 1: 78–94. 10.1111/bjc.12040. 24588763

[erv70053-bib-0010] Kelly, A. C. , J. C. Carter , and S. Borairi . 2014. “Are Improvements in Shame and Self‐Compassion Early in Eating Disorders Treatment Associated With Better Patient Outcomes?” International Journal of Eating Disorders 47, no. 1: 54–64. 10.1002/eat.22196. 24115289

[erv70053-bib-0011] Krippendorff, K. 2019. Content Analysis: An Introduction to Its Methodology. Fourth Edition ed. 10.4135/9781071878781.

[erv70053-bib-0012] Lucre, K. 2022. “Compassion‐Focused Group Psychotherapy for People Who Could Attract a Diagnosis of Personality Disorder.” In Compassion Focused Therapy: Clinical Practice and Applications. Routledge. 10.4324/9781003035879-18.

[erv70053-bib-0013] Marques, C. C. , L. Palmeira , P. Castilho , et al. 2024. “Online Compassion Focused Therapy for Overeating: Feasibility and Acceptability Pilot Study.” International Journal of Eating Disorders 57, no. 2: 410–422. 10.1002/eat.24118. 38124655

[erv70053-bib-0014] Millard, L. A. , M. W. Wan , D. M. Smith , and A. Wittkowski . 2023. “The Effectiveness of Compassion Focused Therapy With Clinical Populations: A Systematic Review and Meta‐Analysis.” Journal of Affective Disorders 326: 168–192. 10.1016/j.jad.2023.01.010. 36649790

[erv70053-bib-0015] Nechita, D.‐M. , S. Bud , and D. David . 2021. “Shame and Eating Disorders Symptoms: A Meta‐Analysis.” International Journal of Eating Disorders 54, no. 11: 1899–1945. 10.1002/eat.23583. 34302369

[erv70053-bib-0016] Vrabel, K. R. , G. Waller , K. Goss , B. Wampold , M. Kopland , and A. Hoffart . 2024. “Cognitive Behavioral Therapy Versus Compassion Focused Therapy for Adult Patients With Eating Disorders With and Without Childhood Trauma: A Randomized Controlled Trial in an Intensive Treatment Setting.” Behaviour Research and Therapy 174: 104480. 10.1016/j.brat.2024.104480. 38310672

