# Supplementary material for: The Acceptability of a Rolling Compassion Focused Therapy Group Embedded Within an Eating Disorder Day Hospital Programme: A Brief Report
Source: Eur Eat Disord Rev. 2025 Nov 6;34(3):557–62. doi: 10.1002/erv.70053 (PMC13048736; doi:10.1002/erv.70053)
Supplement: Supplementary file 1 — Table S1: Findings from quantitative content analysis of open‐ended responses to two questions. [file ERV-34-557-s001.docx]

| **Supplementary File. Table 1. Findings from quantitative content analysis of open-ended responses to two questions.** | | |  |
| --- | --- | --- | --- |
| **Category/description** | **Sub-category** | **Example Quotes** | ***n*** |
| ***Q1. What was most helpful from today's session and why?*** | | |  |
| **CFT Theory: Engaging with the theory and practices of CFT** | - |  | **62** |
|  | Three systems theory | “Learning about the three systems helped me to become aware of them in my life”.  “Recapping three systems – you can learn something new about them every time”.  “Greater awareness of Drive- how it can help and hinder. Useful reminders to check what system I'm in…”. | 14 |
|  | Exploring the definition of compassion | “Learning about the definition of compassion and understanding the qualities/misconceptions better”.  “Learning the qualities of compassion. Realizing it's a two-step process”.  “Learning about compassion and how we can be compassionate with ourselves and other people”. | 9 |
|  | Visual imagery exercise | “I found the calm place imagery quite helpful for self soothing”.  “Vivid imagery felt relaxing and could tune into my imagination and connection to my soothing system”.  “Forming the compassionate companion”. | 9 |
|  | Tricky brain | “Looking at tricky brain loops and being able to separate yourself from them”.  “Learning that the brain is very tricky but not my fault”.  “Learning specifics of our brain, that it's not our fault, how our brains are made up, because it helps to feel like I'm not a bad person for feeling poorly”. | 9 |
|  | Breathing exercise | “The breathing exercises, they really helped me to unwind and mentally relax”.  “Today I just really enjoyed the grounding exercises. Just feel overwhelmed sometimes coming to the end of the week, so found it relaxing”.  “The mindful breathing exercise at the start”. | 7 |
|  | Exploring the self-critic | “It was helpful to address the inner critic to see how it might differ for those with ED/compared to others without”.  “Talking about that inner critical voice and the feelings around it. It helps to understand why I think the way I do”.  “Breaking down the self-critic via the psychologists 'challenging' questions”. | 6 |
|  | Case studies  Using the senses | “I liked using the example of Michaela to talk about EDs and the struggles of…turning your life around”.  “Exercise on Michaela & 3 qualities of compassion & thinking I don't know how to help when & hearing / realizing how others must feel about me”.  “Worksheet on Sarah”.  “Holding the rock”.  “I also liked the stones during the grounding exercise”.  “Collecting different scents to soothe me”. | 5  3 |
| **Group process: Being in group and connecting with others** | - | - | **47** |
|  | Open group discussions | “Talking with the others about their similar experiences.”  “Just hearing that people are kind of experiencing the same thing, sometimes [you] feel so trapped and isolated…but then you hear other group members have similar situations, [and you] don't feel as alone”.  “The open talk of eating disorders really helps me”. | 28 |
|  | Small groups | “Using small groups for activities - felt more connection and people open up more”.  “Smaller group work activities as I didn't feel as intimidated to speak”.  “The smaller group work because it allowed me to connect ore with individual members of the larger group”. | 9 |
|  | Games | “The game, it was a good way for everyone to be included”.  “Found game helpful today because I was very tired so just found it helpful to get up and moving”.  “I liked the “beer pong” activity because it felt more relaxed and an easy way after talking about the self-critic”. | 4 |
|  | Feeling safe | “Being in a safe space to openly talk about my struggles”.  “Feeling safe to open up about my thoughts”.  “The way the session was conducted really helped me feel safe talking about a topic I feel”. | 4 |
|  | Time to speak | “Everyone got a chance to speak. Very interactive- really liked this part”.  “Everybody being given time to speak”. | 2 |
| **Self-awareness: Describing a self-awareness through the sessions** | - | “Awareness and understanding of the ED noise. Focus on sitting with it. We don't need to fix”.  “I connected with myself through others’ words”.  “Asking myself what would be helpful and not harmful for me right now”. | **9** |
| **Unsure what was helpful** | - | “Not too sure can answer that, had a lot of hard time trying to fully concentrate…”.  “Was feeling very anxious before hand contemplated not coming but really glad, I did definitely benefit from it. I still find the group challenging but I think that’s a good thing”. | **2** |
| ***Q2. Any other feedback? (helpful/ unhelpful?) How to improve the session?*** | | |  |
| **Positive feedback: favourable comments** |  |  | **16** |
|  | Overall helpfulness | “One of the most helpful groups in the day programme”.  “Overall, really helpful”.  “Really helpful”. | 5 |
|  | Openness from group | “I really felt the openness from the group and engagement was very good and comforting today”.  “The groups honesty helps it feel like a safe space/and helps you feel supported”.  “Continued open group engagement. Openness about ED behaviours and how to move away by using compassion”. | 5 |
|  | Visual and written materials | “The visuals like the page in front made it easier to connect”.  “The interaction, writing down a mind map was really useful”.  “Visual helps me sit with things”. | 3 |
|  | Space to reflect | “Like today. It is good to stay with the moment and not just move on”.  “Great session, gave me a lot of insight!”.  “Find it very good for grounding me”. | 3 |
| **Engagement issues: barriers to group participation.** | - | - | **12** |
|  | Desire for more group interaction | “It's discouraging/upsetting when people/other members don't want to engage in group”.  “If more people interacted/engaged in group it would be more beneficial”.  “Maybe each person should have to speak once”. | 7 |
|  | Attentional barriers | “Just found it really hard to hear/ concentrate in group activities”.  “Maybe have more breaks, it is hard to concentrate for such a long time”.  “Just a little bit distracting one of the members being outdoors for group…just was a little off putting today”. | 5 |
| **Miscellaneous negative feedback** | - | “Too many meditations and small groups”.  “Found it hard when people were relating to previous sessions because it was my first session”.  “Too much like a learning environment (i.e., school) at the start.”. | **6** |
| **Suggested improvements to the programme** | - | “Talking about how to shut that inner voice down might help. Also work on how to love and accept ourselves…”.  “Keep group in-person”.  “Link the discussion to theory of CFT more”. | **4** |
